# *Pomacea canaliculata* hemocyanin as a novel natural immunostimulant in mammals

**DOI:** 10.3389/fimmu.2024.1490260

**Published:** 2025-01-08

**Authors:** Ignacio Rafael Chiumiento, María Alejandra Tricerri, María Fernanda Cortéz, Santiago Ituarte, Julia Tau, Karina Valeria Mariño, Paola Lorena Smaldini, Horacio Heras, Marcos Sebastián Dreon

**Affiliations:** ^1^ Instituto de Investigaciones Bioquímicas de La Plata “Prof. Dr. Rodolfo R. Brenner”, (INIBIOLP), Universidad Nacional de La Plata (UNLP) - Consejo Nacional de Investigaciones Científicas y Técnicas (CONICET), La Plata, Argentina; ^2^ Cátedra de Química Biológica, Facultad de Ciencias Naturales y Museo, UNLP, La Plata, Argentina; ^3^ Cátedra de Bioquímica Clínica I, Facultad de Ciencias Médicas, UNLP, La Plata, Argentina; ^4^ Cátedra de Bioquímica y Biología Molecular, Facultad de Ciencias Médicas, UNLP, La Plata, Argentina; ^5^ Laboratorio de Glicómica Funcional y Molecular, Instituto de Biología y Medicina Experimental (IBYME), CONICET, Buenos Aires, Argentina; ^6^ Instituto de Estudios Inmunológicos y Fisiopatológicos (IIFP), UNLP - CONICET, La Plata, Argentina

**Keywords:** gastropods, mollusc, respiratory pigment, innate immunity, macrophage

## Abstract

**Introduction:**

Gastropod hemocyanins are potent immunostimulants in mammals, a trait associated with their large molecular size and unusual glycosylation patterns. While the hemocyanin from the marine snail keyhole limpet (KLH), has been widely studied and successfully employed as a carrier/adjuvant in several immunological applications, as well as a non-specific immunostimulant for bladder cancer treatment, few other gastropod hemocyanins have been biochemically and immunologically characterized. In this work, we investigated the immunogenic properties of the hemocyanin from *Pomacea canaliculata* (PcH), an invasive south American freshwater snail. This species, known for its high reproductive rate and easy rearing, represents a promising source of potential biomedical compounds, including hemocyanin.

**Methods:**

Employing flow cytometry, fluorescence microscopy, immunoassays, and quantitative PCR, we analysed the effects of PcH on THP-1 monocytes and their derived macrophages, as well as its ability to induce humoral response on C57BL/6 mice. Additionally, we evaluated the structural stability of PcH across a wide range of temperature and pH values.

**Results and discussion:**

Our findings demonstrate that PcH is a structurally stable protein that not only triggers a pro-inflammatory effect on THP-1 derived-macrophages by increasing IL1-β and TNF-α levels, but also promotes phenotypic changes associated with the monocyte-to-macrophage differentiation. Moreover, the humoral response induced by PcH in mice was indistinguishable from that of KLH, highlighting the promising immunostimulatory properties of this freshwater snail hemocyanin.

## 1 Introduction

Molluscan hemocyanins are large respiratory proteins widely distributed among invertebrates in nature. They are enormous multimeric glycoproteins freely dissolved in the haemolymph, representing up to 90% of the total protein content in this fluid (1). Structurally, these respiratory proteins consist of partially hollow cylinders, composed of ten subunits, each around 400 KDa, associated in di-, tri-, and even multi-decamers. Thus, they are among the largest proteins in nature, with molecular masses ranging from 4 to 8 MDa or even higher. In addition, they exhibit high thermal stability, with melting temperature reaching up to 80°C in some species (2–4). Despite these common structural features, the great mollusc diversity leads to marked differences among groups (5).

In the last decades, immunotherapies have emerged as alternatives to traditional chemotherapies, particularly in developing new antitumoral treatments (6, 7). Notably, molluscan hemocyanins are potent natural immunostimulants in mammals, enhancing both innate and adaptive immune responses with promising clinical outcomes (8, 9). They have been used as carrier proteins coupled to different antigens, including tumour-associated carbohydrate antigens (TACA) (10, 11). They also serve as non-specific immunostimulants in therapeutic cancer vaccines and other anti-tumour strategies, acting as adjuvants to counteract immune self-tolerance to tumour antigens (8, 12). The immunomodulatory effects of hemocyanins are linked to their unique features such as their enormous size which allows for prolonged antigen presentation (8), and their heterogeneous glycosylation patterns (13, 14). These patterns include methylated hexoses, fucose or xylose residues, and truncated N-glycans (14, 15). These glycans interact with immune receptors like C-type lectin receptors and Toll-like receptor 4 on leukocytes, promoting the secretion of Th1-type inflammatory cytokines (16).

The first molluscan hemocyanin employed in biomedical studies was the keyhole limpet hemocyanin (KLH), isolated from the haemolymph of the marine gastropod *Megathura crenulata* (9). KLH has been successfully employed as a non-specific immunostimulant for the treatment of recurrent superficial bladder cancer, with negligible toxic side effects (17), making it an ideal therapeutic agent for long-term continuous treatment. In the last decades, due to the limited bioavailability and growing demand for KLH, there has been a marked interest in identifying and characterizing new hemocyanins with similar or better immunological properties. In this sense, various new gastropod hemocyanins have been studied, mostly from marine species such as RtH from *Rapana thomasiana* (18–20), HtH from *Haliotis tuberculata* (21, 22), CCH from *Concholepas concholepas* (23, 24), FLH from *Fissurella latimarginata* (25), and HaH from the pulmonated *Helix aspersa* (20).

Recently, a hemocyanin was isolated from a freshwater snail, *Pomacea canaliculata* (Caenogastropoda, Ampullariidae). This protein named PcH is a KLH-type hemocyanin organized in di-decamers of 390 kDa subunits (26). The glycan moiety of PcH (2.8% w/w) includes terminal galactose and GalNAc residues, as well as high mannose and complex type N-glycans, containing structures compatible with the T-antigen (27). PcH is of particular interest because it can be purified in large quantities from adult *P. canaliculata*, a South American freshwater snail. This species is especially significant as it is an invasive species with a high reproductive potential and is easy to rear in laboratory conditions. Since its introduction to China in the 1980s, it has rapidly spread worldwide, prompting extensive studies on its biology, physiology, and metabolism (28). These attributes highlight the potential of this species as a source of compounds with promising biomedical use, such as PcH.

In the present work, we study the immunostimulant properties of PcH and evaluate its structural stability to explore its potential application as a bioactive compound. We evaluated the effect of PcH on macrophages focusing on pro-inflammatory cytokines, and the morphophysiological changes induced in a human monocyte leukemic cell line employing flow cytometry, fluorescence microscopy, immunoassays, and quantitative PCR. In addition, we evaluated *in vivo* the immunogenic activity of PcH in C57BL/6 mice measuring the sera IgG titers by ELISA and characterized its structural stability against temperature and extreme pH values by absorption and fluorescence spectroscopy.

## 2 Materials and methods

### 2.1 Animals

Adult *P. canaliculata* snails were collected in water streams near La Plata city, Buenos Aires province, Argentina (40° 42´ 46´´ S, 74° 0´ 21´´W), genetically identified by sequencing a cytochrome C oxidase I gene fragment by using LCO1490 and HCO2198 primers (29), and reared in the laboratory. All studies followed the legislation of the Argentinean provincial Wildlife Hunting Law (Ley 5786, Art. 2).

Six-week-old female C57BL/6JLAE mice were obtained from the Experimental Animals Laboratory of the School of Veterinary Science (UNLP, Argentina), housed in the facilities of the School of Medicine (UNLP, Argentina), at 22°C with light/dark cycle of 12/12 h and fed *ad libitum* with sterile food and water.

Studies complied with the Guide for the Care and Use of Laboratory Animals (30) and were approved by the “Comité Institucional de Cuidado y Uso de Animales de Experimentación” (CICUAL) of the School of Medicine, UNLP (P02-01-2024).

### 2.2 Protein sample preparation, NaIO_4_ treatment, and de-N-glycosylation

Lyophilized KLH was purchased from Sigma Chemical Co. (Cat. No. H7017), reconstituted in phosphate buffer saline (PBS) according to the manufacturer’s guidelines and sterilized by 0.22 µm filtration. Native PcH was isolated from the haemolymph collected through exsanguination of 6 adult snails under sterile and pyrogen-free conditions. The purification of PcH was performed as previously described (27). Briefly, samples were pooled, and cell free haemolymph was obtained by sequential centrifugation at 500 x g, 10 min and 10,000 x g, 10 min. The obtained supernatant was layered on a tube containing NaBr (δ =1.28 g/mL) and centrifuged at 200,000 x g, 22 h, in a swinging bucket rotor SW60.Ti on a Beckman L8M (Beckman, Palo Alto, CA). After centrifugation, PcH-containing fractions -blue color bands- were collected and pooled to further purify the protein by size exclusion liquid chromatography on a Superose-6 column (Amersham-Pharmacia, Uppsala, Sweden) coupled to an Agilent 1260 HPLC system (Agilent Technologies) using two different mobile phases, PBS or 20 mM Tris-HCl pH 7.4, 20 mM CaCl_2_ and MgCl_2_ buffer, as appropriate for the experiments. Deglycosylated PcH forms were obtained by an oxidative procedure incubating with 15 mM sodium periodate for 1 h in the dark at room temperature as previously described (31). PcH was also de-N-glycosylated by treatment with glycerol-free PNGase F (New England Biolabs) using dissociating conditions to give better access to oligosaccharides, as previously described by Salazar and coworkers (32). Briefly, PcH was dialyzed against dissociation buffer (130 mM glycine containing 2.5 mM EDTA, pH 8.6), and then heated at 60 or 100°C for 15 min. PcH was then cooled to room temperature, and 2 μl of PNGase F in deionized water was added and incubated for 24 h at 37°C. After deglycosylation, both PcH samples were washed with PBS and concentrated to 1 mg/mL employing an Amicon Ultra-15 50K MWCO device (Millipore). Glycan removal was verified through PAGE analysis, by comparing the electrophoretic pattern of the deglycosylated PcH forms with the negative control for each treatment. The protein content of PcH preparations was determined spectrophotometrically at 280 nm and their purity was checked using PAGE. For cell culture assays, hemocyanin samples were sterilized by filtration through 0.22 µm membranes and endotoxin levels were determined spectrophotometrically, following the method described by Karkhanis and coworkers (33).

### 2.3 PcH structural stability against pH and temperature

To evaluate the influence of pH on the protein structure, 1.0 mg/mL PcH at pH values ranging from 2.0 to 12.0 were prepared. Buffers were formulated using 100 mM sodium phosphate salts, except for pH 4.0 buffer which was prepared by mixing 100 mM sodium citrate and 200 mM Na_2_HPO_4_ 0.2 M. Samples were incubated for 48 h at 4°C in the dark and analysed by fluorescence and absorption spectroscopy at 25°C.

To assess thermal stability, 0.6 g/L PcH dissolved in 20 mM Tris-HCl buffer (pH 7.4) containing 10 mM CaCl_2_ and 10 mM MgCl_2_ was subjected to rising temperatures from 25 to 85°C. Once the desired temperature was stable, fluorescence and absorption spectra were acquired.

#### 2.3.1 Absorption spectroscopy

UV-visible spectra were recorded between 250 and 310 nm using an Agilent 8453 diode array spectrophotometer (Agilent Technologies, Waldbronn, Germany). The temperature of the sample holder was controlled using a circulating water bath (Lauda-Königshofen, Germany). For each sample, three spectra were acquired and averaged, and its corresponding buffer was subtracted. In all cases, the fourth derivative spectra were calculated.

#### 2.3.2 Fluorescence spectroscopy

Intrinsic fluorescence spectra of PcH at different temperatures and pH values were recorded in emission scanning mode in a Varian Cary Eclipse spectrofluorometer (Varian Inc., Australia). Tryptophan residues were excited at 290 nm (5 nm slit) and the emission was recorded between 310 and 410 nm (5 nm slit) in a 1 cm optical path quartz-cell. The temperature of the sample holder was controlled by employing a circulating water bath (LAUDA, Lauda-Königshofen, Germany). For each sample, three emission spectra were acquired, averaged, and corrected for buffer fluorescence. In all cases, the mass center was calculated.

### 2.4 Cell culture

The human pro-monocyte cell line THP-1 was obtained from ECACC (Salisbury, UK). Cells were cultured in RPMI 1640 medium (Serendipia Lab, Buenos Aires, Argentina) with 25 mM sodium bicarbonate, 25 mM Hepes, pH 7.3 supplemented with 10% v/v fetal bovine serum (FBS, NATOCOR, Villa Carlos Paz, Córdoba, Argentina), and 100 nM penicillin/streptomycin (Life Technologies, Grand Island, NY) at 37°C with 5% CO_2_ in a humidified atmosphere. The cellular density was kept below 5 x 10^5^/mL by diluting the cultures with fresh medium every 5 to 7 days.

#### 2.4.1 Pro-inflammatory effects of PcH

To promote macrophage transformation, THP-1 cells were seeded on a 24-well plate at approximately 10^6^ cells/mL (0.5x10^6^ cells/well), and exposed to 5 ng/mL phorbol 12-myristate 13-acetate (PMA, Sigma Chemical Co.), for 48 h. Once transformed, the PMA-containing medium was removed, and the macrophages were washed twice with PBS and cultured for 24 h in fresh medium. Finally, cells were exposed for 3 h to PcH as well as to its de-glycosylated forms, at concentrations ranging from 0.015 to 1.000 g/L in PBS, employing 50 ng/mL of lipopolysaccharide (LPS, Sigma Chemical Co.) and PBS as positive and negative control of the assay, respectively. After treatments, cell viability was evaluated by 3-[4,5-dimethylthiazol-2-yl]-2,5-diphenyltetrazolium bromide (MTT, Sigma Chemical Co.) assay as we previously described (34) and the levels of TNF-α and interleukin-1β were determined in the culture supernatants by ELISA (BD Biosciences, San Diego, CA) as previously described (35).

#### 2.4.2 Effect of PcH on monocyte differentiation

To evaluate the ability of PcH to promote THP-1 cell differentiation to macrophages, THP-1 cells were seeded on multiwell plates at approximately 10^6^ cells/mL unless otherwise stated and incubated with 1 g/L of the purified protein in PBS buffer. The phenotypic changes observed were measured and compared with 5 ng/mL of PMA and PBS as positive and negative controls, respectively. Experiments were performed by triplicates and cell morphology, cell adhesion, and gene differential expression of different macrophage markers were analysed as described below.

##### 2.4.2.1 Cell adhesion

Cell adhesion was evaluated employing the Hoescht-33258 fluorophore. Briefly, cells were seeded on 96-multiwell plates and incubated for 24 h either in the presence or absence of PcH, as described above. Culture supernatants were centrifugated at 500 x g, 10 min and the pelleted cells were washed with RPMI 0.5% FBS, the obtained pellet was resuspended in distilled water and incubated for 15 min in the dark at 37°C with 100 μL of Hoescht-33258 (Sigma Chemical Co.), 0.02 mg/mL in TNE buffer (Tris-HCl 10 mM, pH=7.4, NaCl 2 M, EDTA 1 mM). Well-adhered cells were treated similarly. Sample fluorescence was registered at 460 nm (λex, 358 nm) in a multiwell reader DTX 880 (Beckman Coulter, CA, USA). In addition, images of adhered and non-adhered cells were acquired in an inverted fluorescence microscope (Olympus IX-71, Tokyo, Japan).

##### 2.4.2.2 Flow cytometry

THP-1 monocyte cells were seeded in a multi-12 well plate and incubated for 72 h in RPMI 0,5% FBS with PcH 1 mg/mL, PBS or PMA 5 ng/mL. The culture supernatant, containing the non-adhered cells, was taken and the adhered cells were gently scrapped and resuspended in fresh medium. Both cell populations of each treatment were pooled, washed once, and resuspended in PBS, and their cell size and granularity were inferred by the forward (FSC) and side scattered (SSC) light respectively. Additionally, to further evaluate this differentiation process, the same experiment was performed and, before harvesting, cells were incubated for 15 min at 37°C with 1 nM MitoTracker DeepRed (Invitrogen, Waltham, MA, USA), a mitochondrial-potential dependent dye, dissolved in RPMI without neither phenol red nor FBS.

The assays were performed on a BD AccuriTM C6 Plus Flow Cytometer (BD Bioscience, San Diego, CA, USA), with a two-laser base configuration: 488 nm solid-state blue laser and 640 nm diode red laser, carrying standard optical filters: FL1 533/30 nm, FL2 585/40 nm, FL3 >670 nm and FL4 675/25 nm. The latter was employed for detecting the emission of MitoTracker DeepRed excited by the 640 nm laser. Cells solely incubated with RPMI were employed as autofluorescence control and subtracted to the median fluorescence intensity (MdFI) of each treatment. For each sample 50,000 events were recorded.

Data transformation and analysis were performed using the FlowJo™ X 10.0.7r2 Software (BD Life Sciences). To define populations, the FSC-A vs SSC-A gate was used for all data files. Experiments were performed by triplicates or quadruplicates.

##### 2.4.2.3 Quantitative RT-PCR

THP-1 cells were seeded in 60 mm culture plates at a density of 2.5 x 10^6^ cells/mL (2.5 x 10^6^ cells/plate) and cultured for 36 h in the presence of 1 g/L of PcH and 5 ng/mL of PMA. Total RNA was isolated from cultures using TRIzol reagent (Thermo Fisher Scientific Inc.) following the manufacturer’s instructions. The absence of genomic DNA as well as RNA integrity was checked in a 1% agarose gel electrophoresis and total RNA content was quantified at 260 nm in a Nanodrop 2000/2000c (Thermo Fisher Scientific Inc.). Synthesis of cDNA was performed using the iScript cDNA Synthesis Kit (BIO-RAD Laboratories, Inc., Hercules, CA, USA) following the manufacturer’s instructions. Real-time PCR was performed in a AriaMix (Agilent Technologies, Inc) thermocycler using a qPCR iTaq Universal SYBR Green Supermix, (BIO-RAD Laboratories, Inc., Hercules, CA, USA) to determine the relative gene expression levels in each sample employing the following quantitation formula:


$$\text{Ratio}=\frac{{\left(E_{TARGET}\right)}^{\Delta CqTARGET(CALIBRATOR-SAMPLE)}}{{\left(E_{REFERENCE}\right)}^{\Delta CqREFERENCE(CALIBRATOR-SAMPLE)}}$$

where *E* is the qPCR efficiency for each gene, calculated in the corresponding calibration curves as 10^(1-SLOPE REGRESSION LINE)^-1, *TARGET* means each of the markers which expression was evaluated, *REFERENCE* is the housekeeping gene employed, *i.e.* GAPDH, and *CALIBRATOR* the PBS-treated cells used as negative control.

The oligonucleotide primer sequences for well-characterized macrophage markers, including CD-80, CD-86, iNOS, CD-206, CD-263, and CD-11b, as well as GAPDH used as housekeeping gene, are listed in **Table 1**.

**Table 1:** Oligonucleotide primer sequences employed to evaluate the differential expression of macrophage markers.

	GENE	FORWARD PRIMER	REVERSE PRIMER
Pan Macrophage	CD68	CGAGCATCATTCTTTCACCAGCT	ATGAGAGGCAGCAAGATGGACC
	CD11b	GGAACGCCATTGTCTGCTTTCG	ATGCTGAGGTCATCCTGGCAGA
M1	CD86	CCATCAGCTTGTCTGTTTCATTCC	GCTGTAATCCAAGGAATGTGGTC
	CD80	CTCTTGGTGCTGGCTGGTCTTT	GCCAGTAGATGCGAGTTTGTGC
	iNOS	GCTCTACACCTCCAATGTGACC	CTGCCGAGATTTGAGCCTCATG
M2	CD163	CCAGAAGGAACTTGTAGCCACAG	CAGGCACCAAGCGTTTTGAGCT
	CD206	AGCCAACACCAGCTCCTCAAGA	CAAAACGCTCGCGCATTGTCCA
Reference	GAPDH	GAGTCAACGGATTTGGTCGT	TTGATTTTGGAGGGATCTCG

##### 2.4.2.4 Quantitation of CD-68 by Western blot

THP-1 cells were seeded in 12-well plates and incubated with 1 g/L of purified PcH in PBS for 72 h. Negative and positive controls were obtained by incubating cells with PBS and 5 ng/mL PMA, respectively. Plates were centrifugated at 500 x g for 10 min, the supernatant discarded, and the cell culture total proteins extracted with 40 µL of RIPA lysis buffer and quantified by Bradford (36). Then, 5 μg of each protein sample was resolved in a 4-12% SDS-PAGE gel and transferred onto a nitrocellulose membrane at 100 V for 1 h in a Transblot Cell (Bio Rad Laboratories, Inc.), using 25 mM Tris–HCl, 192 mM glycine, 20% (v/v) methanol, pH 8.5 buffer. After transfer, membrane was blocked with 5% (w/v) non-fat dry milk in PBS–Tween 0.05% (v/v) for 1 h, and then incubated overnight at 4°C with an anti-CD68 mouse monoclonal antibody diluted 1/10,000 in 1% (w/v) non-fat dry milk in PBS–Tween. After washing with PBS–Tween, the membrane was incubated with goat anti-mouse IgG horseradish peroxidase conjugate (BioRad Laboratories, Inc.) diluted 1/20,000. Immunoreactivity was visualized by ECL in a ChemiDoc Imaging System (BioRad Laboratories, Inc.).

### 2.5 Humoral immunity bioassays

To evaluate the immunogenicity of PcH *in vivo*, groups of three mice were inoculated intraperitoneally with 100 μL of either KLH (Sigma-Aldrich) or PcH, both at 2 g/L in PBS. A third group received the same volume of PBS. Fifteen days later, the immunization was repeated, and ten days after that, mice were euthanized by cervical dislocation and bled by cardiac puncture.

The specific anti-PcH IgG levels in the sera were determined by ELISA. Briefly, 96-well plates (NUNC MaxiSorp, Thermo Scientific) were coated with 100 μL/well at 25 μg/mL of either PcH or KLH in carbonate buffer (pH 9.6) overnight at 4°C. The plates were then washed three times with PBS–Tween 0.05% (v/v) and blocked with 200 μL/well of 3% (w/v) non-fat dry milk in PBS for 1 h at 37°C. After blocking, plates were incubated with 100 µL/well of two-fold serial dilutions of the obtained sera in PBS, 1% (w/v) non-fat dry milk, for 1 h at 37°C. Then, plates were washed and incubated with anti-mouse IgG HRP (BioRad Laboratories, Inc.) (1/10,000 dilution in 1% w/v non-fat dry milk in PBS) 1 h at 37°C. Finally, the plates were washed again and developed by adding 100 µL/well of 1 mg/mL o-phenylenediamine dihydrochloride (OPD) in citrate buffer with 1 μL of H_2_O_2._ The colorimetric reaction was stopped by adding 50 µL/well of 2N H_2_SO_4_ and the OD was measured at 492 nm in a Varioskan LUX reader (Thermo Scientific).

### 2.6 Statistical analysis

Unless otherwise stated, significant differences among samples were evaluated by the One-way ANOVA test using GraphPad Prism software (GraphPad software, Inc., San Diego, CA). Results shown are representative of at least three independent assays.

## 3 Results

### 3.1 PcH structural stability

**Figure 1:**
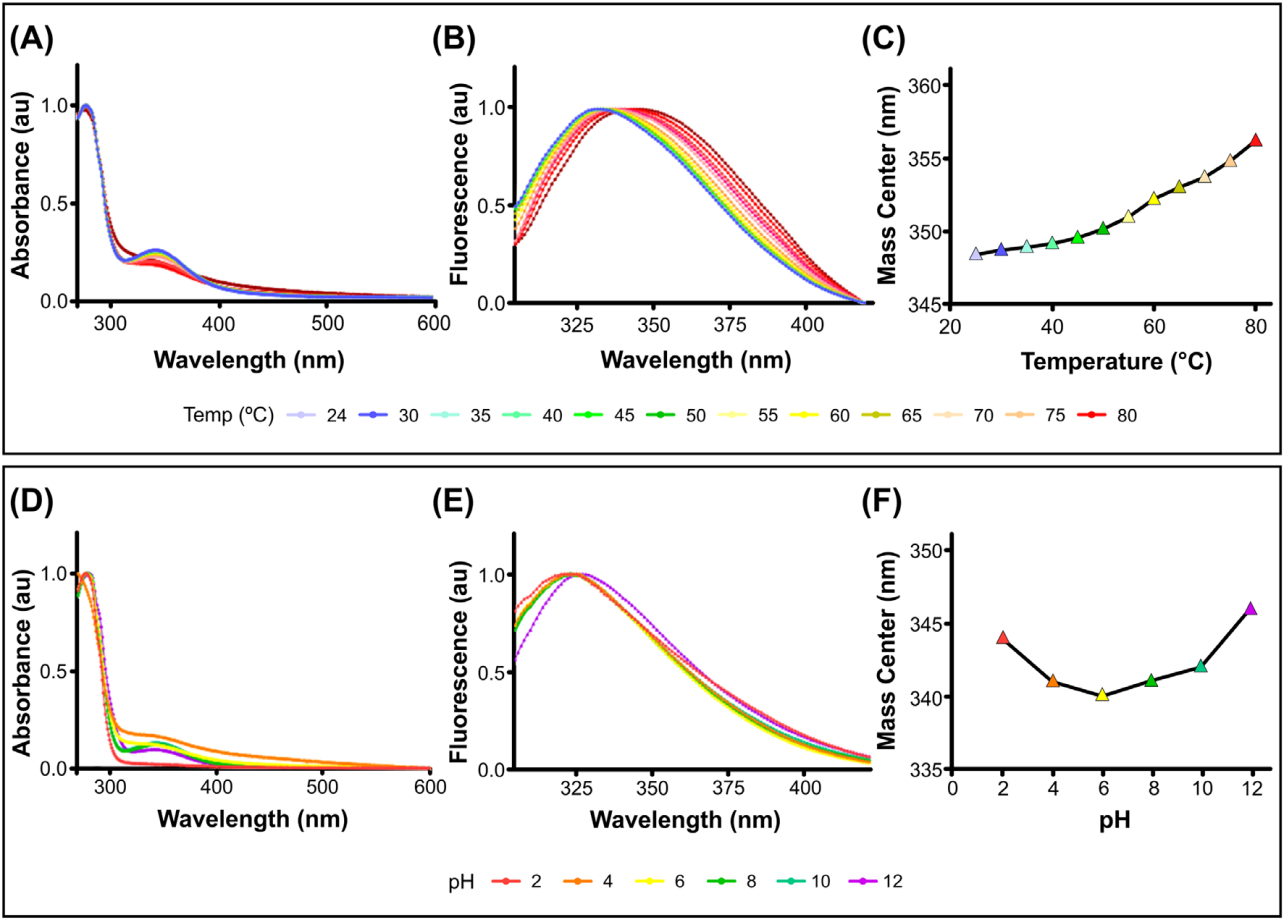
Structural stability of PcH. **(A)** UV-vis absorption spectra, **(B)** normalized intrinsic fluorescence spectra, and **(C)** their mass centers of PcH at different temperatures. **(D)** UV-Vis absorption spectra, **(E)** normalized intrinsic fluorescence spectra, and **(F)** their mass centers of PcH at different pH values.

The structural stability of PcH was evaluated across a wide range of temperatures and pH values by absorption and fluorescence spectroscopy. The protein showed high thermal stability, with absorption spectra showing no perturbation until 80°C (**Figure 1A**), where a shift to higher wavelengths in the corresponding fourth derivatives was observed (**Supplementary Figure 1SA**). In contrast, fluorescence spectra of PcH showed a red shift in their emission maxima as well as an increase in the spectra mass center above 60°C (**Figures 1B, C**), indicative of a slight structural perturbation at this temperature. Regarding pH structural stability, PcH remained stable across a range of pH from 4.0 up to 10.0, as evidenced by both absorption (**Figure 1D**; **Supplementary Figure 1SB**) and fluorescence spectroscopy (**Figures 1E, F**).

### 3.2 PcH cytotoxicity

**Figure 2:**
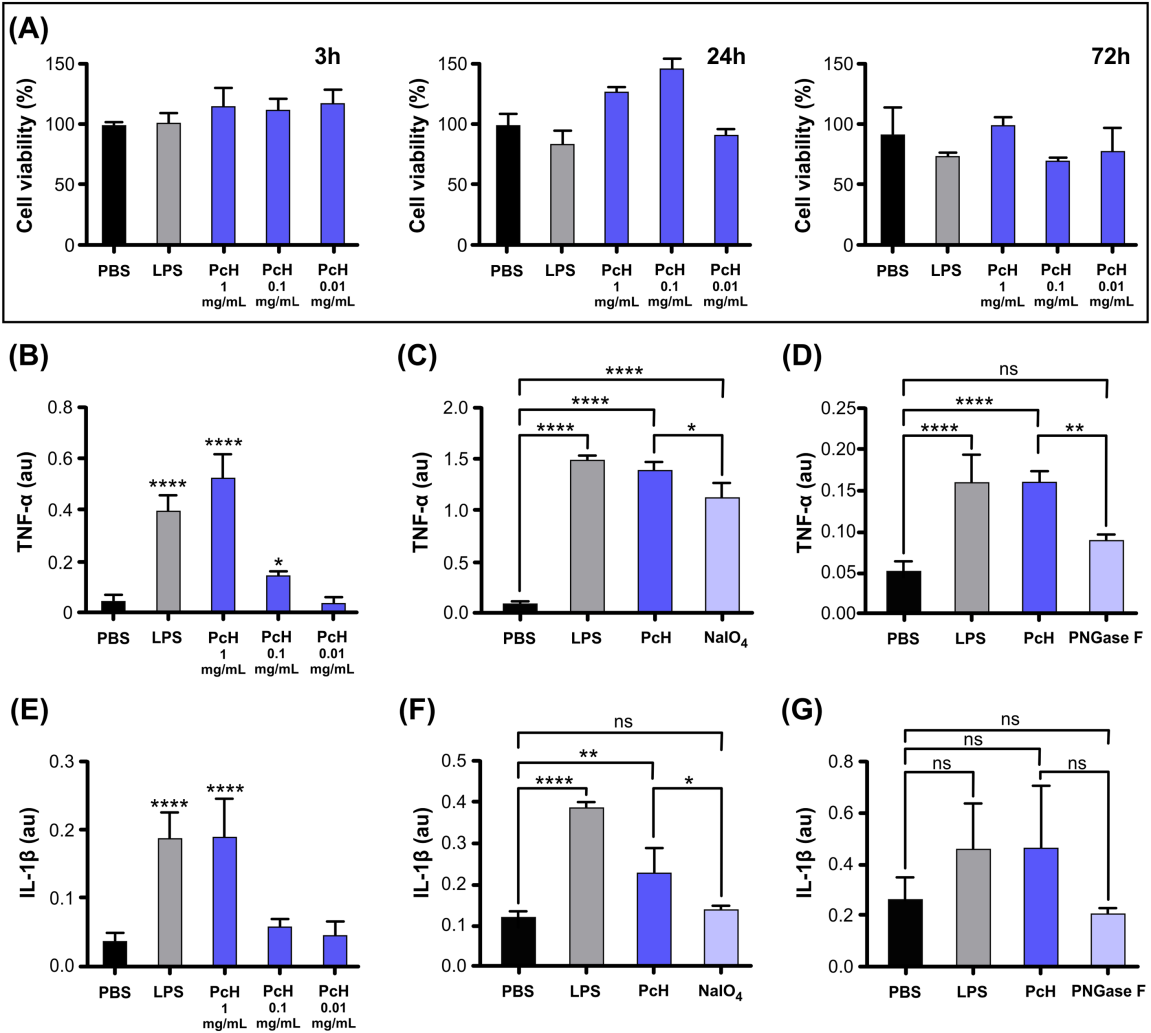
PcH cytotoxicity and induced cytokine secretion profiles on THP-1 derived macrophages. **(A)** Cell viability in THP-1 derived macrophages assessed by MTT assay after 3h, 24h, and 72h exposure to 1, 0.1, and 0.01 mg/mL of PcH. **(B)** TNF-α secretion levels induced by 1, 0.1 and 0.01 mg/mL of PcH, **(C)** 1 mg/mL NaIO4 oxidized PcH, and **(D)** 1 mg/mL de-N-glycosylated (PNGase F) PcH, after 3h exposure. **(E)** IL-1β secretion levels induced by 1, 0.1 and 0.01 mg/mL of PcH, **(F)** 1 mg/mL NaIO4 oxidized PcH, and **(G)** 1 mg/mL de-N-glycosylated (PNGase F) PcH, after 3h exposure. Bars represent the mean ± SD of 3 independent determinations, au: arbitrary units. *P<0.05; **P<0.01; ****P<1x10^-4^; ns, non-significant differences.

The cell viability of THP-1 monocytes was assessed by MTT assay after exposure to different PcH concentrations, revealing a lack of cytotoxic effect after 3, 24, and 72 h of incubation (**Figure 2A**). The endotoxin content in hemocyanin stock preparations was undetectable in our experimental conditions.

### 3.3 Pro-inflammatory response

The pro-inflammatory effect of PcH on THP-1 differentiated into macrophages was confirmed by the significant increase in TNF-α and IL-1β levels in culture supernatants in a dose-dependent manner (**Figures 2B, E**). This cytokine secretion pattern is consistent with a Th-1 response previously observed for other gastropod hemocyanins (12, 24). Interestingly, a significant decrease in TNF-α secretion was observed when PcH was pre-treated with sodium periodate or enzymatically de-N-glycosylated (**Figures 2C, D**, respectively). Regarding IL-1β secretion pattern, a significant decrease was only observed in macrophages exposed to periodate-treated PcH (**Figure 2F**), while a marked, but not significant, decrease was observed for PcH enzymatically de-N-glycosylated (**Figure 2G**). Deglycosylation of PcH was confirmed using 6% native PAGE, which showed a shift in the electrophoretic migration pattern following both treatments (**Supplementary Figure S2**). These shifts are consistent with observations reported for other hemocyanins (23, 32, 37).

### 3.4 Monocyte differentiation

#### 3.4.1 Morphological changes

**Figure 3:**
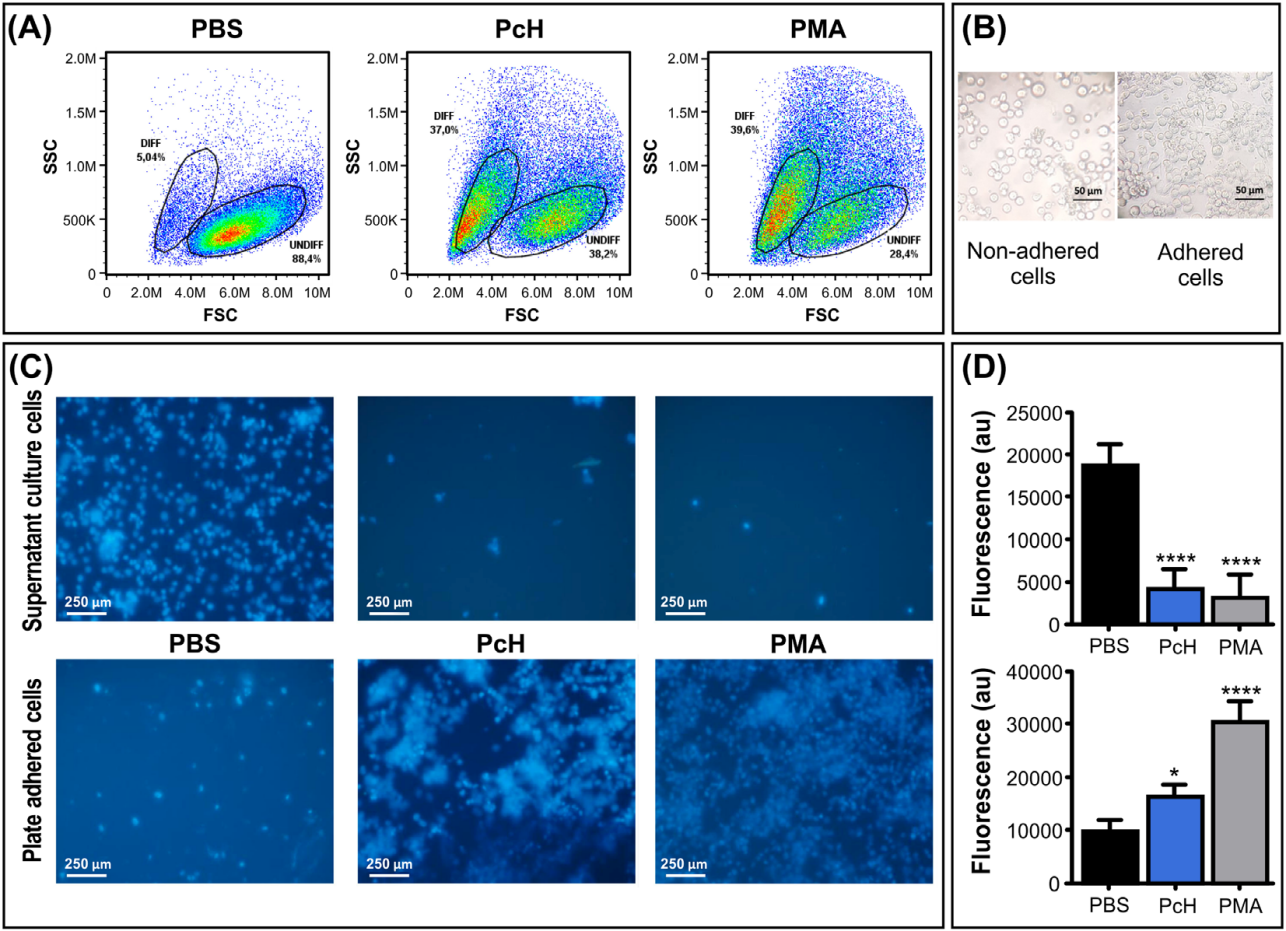
Morphological changes of monocytes upon PcH exposure. **(A)** Forward (FSC) and side light scatter (SSC) plots of THP-1 cells incubated with 1mg/mL of PcH for 72h. Gates: DIFF, differentiated monocytes; UNDIFF, undifferentiated monocytes. Representative data of 3 independent analyses. A total of 50,000 events per sample were acquired. PBS and 5 ng/mL of PMA were used as negative and positive controls, respectively. **(B)** Representative phase contrast photographs, and **(C)** fluorescence microscopy images of adhered and non-adhered THP-1 cells after 24h exposures to 1 mg/mL of PcH employing the nuclear-dying agent Hoescht-33258. **(D)** Total fluorescence intensity of the dye, au: arbitrary units. Bars represent the mean ± SD of 3 independent determinations for each treatment. ****P<1x10^-4^; *P<0.05.

To track the phenotypic changes during the monocyte into macrophages differentiation, the morphological changes in THP-1 human monocytes associated with PcH exposure were determined by flow cytometry. The forward vs. side scattered light dot plots (**Figure 3A**) revealed the rise of a subpopulation with increased subcellular complexity and a reduction in cell size in monocytes treated with PcH compared to PBS-treated cells. These morphological changes are consistent with the effects observed with PMA treatment, which was used as a positive differentiation control.

#### 3.4.2 Changes in cellular adhesion

Monocytes exposed to PcH underwent a phenotypic alteration from suspension-growing cells to adhered ones, as expected for THP-1 macrophage differentiation (**Figure 3B**). To monitor cellular behaviour, THP-1 nuclei were stained with the Hoescht-33258 dye which significantly increases its quantum yield when bound to DNA. Fluorescence microscopy observations suggested a higher number of cells adhered to the plate when treated with PcH or PMA compared to treatment with PBS alone (**Figure 3C**). The fluorescence quantification in each condition confirmed significant differences in the suspended/adhered cell ratio upon PcH treatment (**Figure 3D**).

#### 3.4.3 Metabolic changes

**Figure 4:**
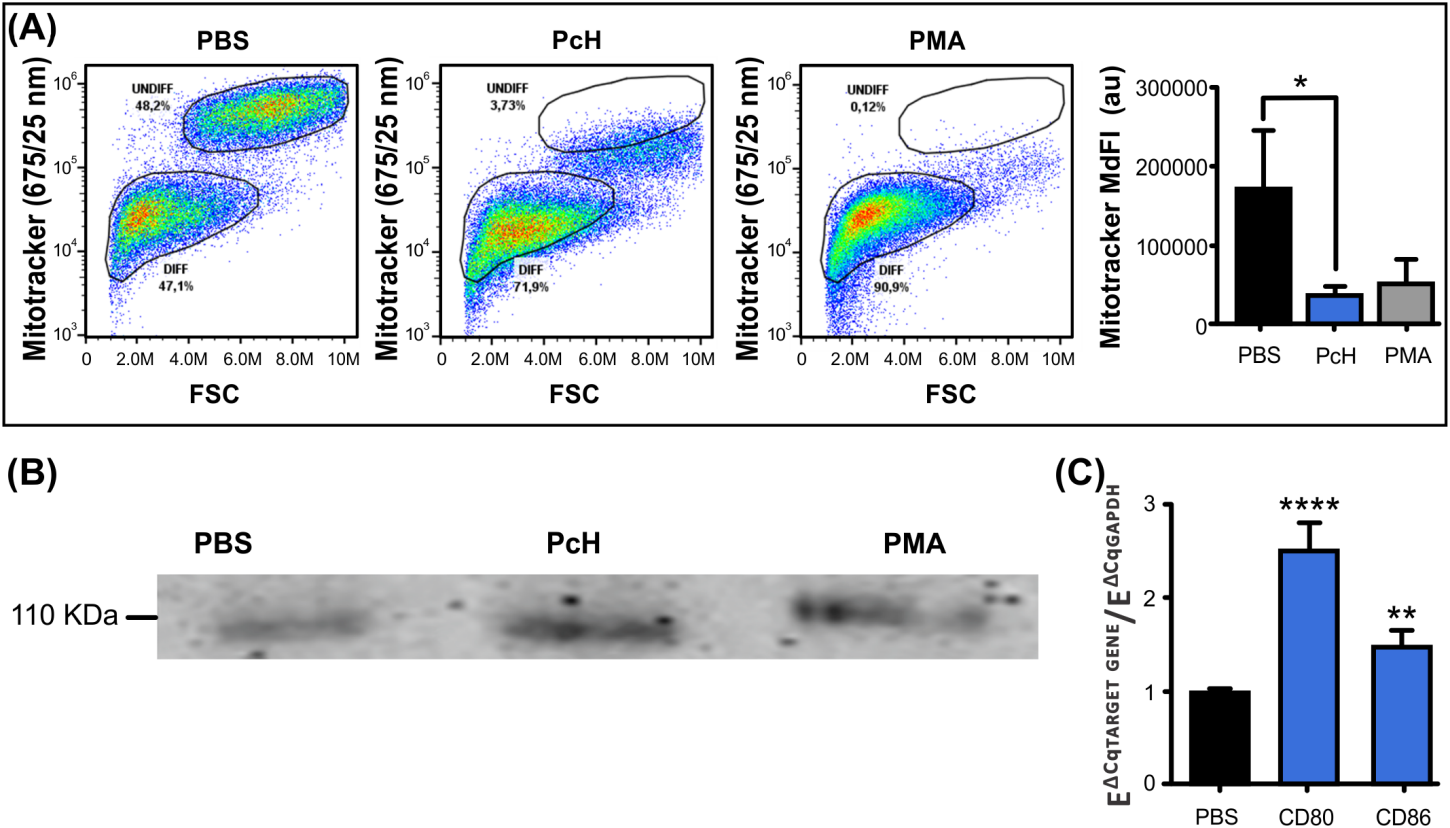
Immunometabolic changes of monocytes upon PcH exposure. **(A)** Metabolic changes on THP-1 monocytes after 1mg/mL PcH exposure as observed in MitoTracker Fluorescence vs. FSC dot plots. Gates: DIFF, differentiated monocytes; UNDIFF, undifferentiated monocytes. Median fluorescence intensity (MdFI) of the fluorescent probe were analysed by Kruskal-Wallis test, au: arbitrary units. Bars represent the mean ± SD of 3 independent determinations for each treatment. *P<0.05. A total of 50,000 events per sample were acquired. PBS and 5 ng/mL of PMA were used as negative and positive controls, respectively. **(B)** The pan macrophage marker CD68 detected by Western Blot on THP-1 cells after PcH exposure. **(C)** Differential gene expression of CD80 and CD86 M1 macrophage markers by qPCR upon PcH exposure, PBS was used as negative control. Bars represent the mean ± SD of 3 independent determinations for each treatment. ****P<1x10^-4^, **P<0.01.

Mitochondrial metabolism is highly dependent on the type of cellular differentiation that monocytes undergo. The monocyte subpopulation with smaller size, as described above, also showed a diminished MitoTracker Deep Red fluorescence intensity (**Figure 4A**), suggesting that differentiating monocytes went through characteristic metabolic changes after PcH exposure. Indeed, monocytes exposed to PcH displayed a decrease in the MitoTracker MdFI compared to PBS-exposed monocytes, which is consistent with the effects observed for PMA-exposed monocytes. Such a reduction in MdFI is indicative of mitochondrial membrane hyperpolarization and a metabolic shift towards a glycolytic phenotype, as it is expected for proinflammatory M1 macrophage differentiation.

#### 3.4.4 Macrophage differentiation markers

To confirm the macrophage differentiation of PcH-exposed monocytes, we analysed the presence of the pan-macrophage marker CD68 by Western blot. Monocyte cultures showed an increase in CD-68 for PcH and PMA-treated cells as shown in **Figure 4B**. To further characterize the effect of PcH on monocyte differentiation, we evaluated the differential gene expression of M1/M2 macrophage markers by qPCR. We found a significant increase in the expression levels of two M1 markers, namely CD86 and CD80, after 36 h exposure to PcH (**Figure 4C**). No significant changes were observed for the M2 markers employed (not shown).

### 3.5 *In vivo* humoral response

**Figure 5:**
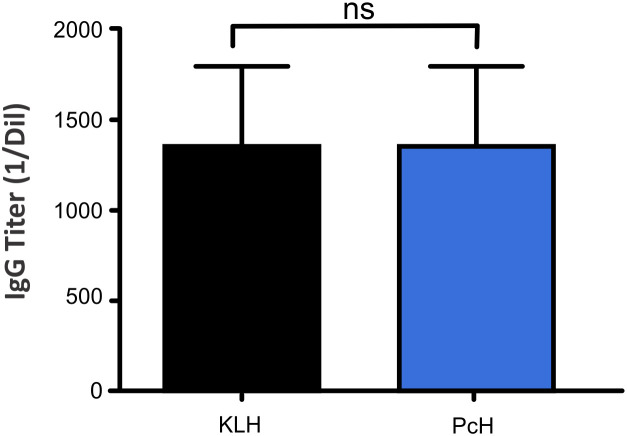
Mice humoral response to PcH. Serum IgG titers of mice immunized with PcH were compared with those immunized with KLH determined by ELISA. Bars represent the mean ± SD of 3 independent determinations for each hemocyanin. ns, non-significant differences.

The immunogenicity of PcH was evaluated *in vivo* in C57BL/6 mice. The IgG titers in the sera of PcH and KLH immunized animals were determined by ELISA, averaged and the PBS-group titer was subtracted. Notably, not-significant differences in the IgG titers between both groups of mice were observed, indicating that both, PcH and KLH trigger a similar humoral response (**Figure 5**).

## 4 Discussion

Gastropod hemocyanins have rapidly gained research interest due to their immunomodulatory properties, making these glycoproteins attractive candidates for biomedical applications. Although only KLH is currently used therapeutically, a few other marine gastropod hemocyanins have shown promising results (8). No recombinant hemocyanin has been obtained, making its production entirely dependent on purification from marine snail sources. In this work, we studied the immunostimulant capacity of a freshwater gastropod hemocyanin for the first time and evaluated its structural stability across a wide range of pH and temperatures. Our results expand the knowledge on molluscan hemocyanins, providing insights into their immunogenic mechanism and potential biomedical applications.

The structural stability of PcH was evaluated in a wide range of temperature and pH values. While the protein does not completely unfold at 80°C, similar to reports for *Helix aspersa* and *H. lucorum* hemocyanins with Tm values of 79.8°C and 82.3°C, respectively (2, 4), slight structural perturbations were observed above 55°C. Interestingly, PcH remains stable across a broader range of pH values than that reported for *H. lucorum* hemocyanin (4). This structural stability of PcH in a wide range of pH and temperature values highlight the versatility of this hemocyanin as an immunostimulant molecule.

We verified the pro-inflammatory effect of PcH, through increased cytokine levels of IL1-β and TNF-α after exposing THP-1-derived macrophages to PcH. This cytokine secretion pattern is strongly associated with Th-1 response (38) and agrees with that observed for KLH (24). Previously, Yasuda and Ushio (39) found that KLH activates the inflammation-related transcription factor NF-κB in THP-1 cells, while Zhong and collaborators (40) demonstrated that marine gastropod hemocyanins promote the differential gene expression of proinflammatory cytokines, including IL-1β and TNF-α in murine macrophages. In this work, macrophages exposed to deglycosylated PcH (both by oxidation with sodium periodate and by PNGase F digestion) showed significantly decreased secretion levels of TNF-α. In contrast, a significant decrease in IL-1 β secretion was observed only for the periodate deglycosylated form of PcH, suggesting that carbohydrate moieties not removed by PNGase F digestion are important players in the immunomodulatory properties of PcH. These findings are consistent with previous studies on KLH, CCH, and FLH (32, 41), highlighting the relevance of glycosylation in the immunogenic effect of molluscan hemocyanins. The high immunogenicity of native PcH may be linked to the presence of unusual glycan structures as seen in other hemocyanins (5, 15, 42–44), including the Galβ (1–6)Man unit in KLH (13) and a truncated N-glycosylation pattern in CCH (14). In particular, Galβ(1–3)GalNAc (9), an epitope cross-reactive with the Thomsen-Friedenreich (Tf) antigen [a truncated O-glycan structure that is highly overexpressed in epithelial carcinomas (45)] in KLH has been demonstrated to be immunogenic and proposed as a mediator of the beneficial effect of KLH in bladder cancer (46). Notably, the presence of Galβ (1–3)GalNAc has also been reported in PcH by lectin assays (27).

We further studied the effect of PcH on innate immunity by evaluating the phenotypic changes in THP-1 monocytes after PcH exposure. The appearance of a subpopulation with higher cellular complexity and smaller size, together with an increase in cellular adherence was observed in monocytes exposed to PcH and PMA. These phenotypic changes observed in both treatments are indicative of a monocyte-to-macrophage differentiation process (47). Moreover, a metabolic shift to a glycolytic phenotype, associated with ROS production in an M1 pro-inflammatory activation (38, 48), was also observed in monocytes exposed to PcH and PMA. The differentiation of THP-1 monocytes to macrophages was confirmed by the presence of macrophage markers in PcH-exposed monocytes, including CD68, a well-known pan macrophage marker (49, 50), and the increased differential gene expression of CD80 and CD86, indicative of M1 polarization (51). Finally, the ability to elicit a strong humoral response seems to be another key feature of the immunostimulation induced by hemocyanins (9, 31). Native PcH was able to elicit IgG levels like those found with KLH in an *in vivo* assay, highlighting the immunogenic capability of this freshwater gastropod hemocyanin.

Based on our findings and data from other hemocyanins (9, 20, 31, 52), we propose a potential mechanism for the immunomodulatory effects of PcH. It may activate macrophages at specific sites, triggering the release of pro-inflammatory cytokines, while promoting M1 differentiation in newly recruited monocytes. This process likely involves the interaction of PcH glycan moieties with innate immune receptors, such as TLR-4 and C-type lectin (53–55), leading to a Th-1 response that helps to overcome the immune tolerance set up by tumors and other pathologies. This mechanism aligns with the complex structure and glycosylation pattern of PcH (27). Moreover, the very low cross-reactivity of CCH and FLH with anti-PcH polyclonal antibodies (27) suggests that PcH contains unique glycan-related epitopes with potentially different immune properties.

In this scenario, PcH emerges as a promising new immunostimulant. Derived from a well-studied freshwater gastropod, with available genomic and transcriptomic data (56, 57), PcH displays remarkable structural stability across a wide range of temperatures and pH values, expanding its potential for therapeutic applications. Given the lack of recombinant forms and the limited bioavailability of other hemocyanin sources, an easy-to-rear species with a high reproductive rate represents a promising alternative. These findings encourage further investigations into the biochemical and immunological properties of PcH, which may uncover new biomedical applications for this molluscan hemocyanin.

## Data Availability

The original contributions presented in the study are included in the article/**Supplementary Material**. Further inquiries can be directed to the corresponding author.
