# Supplementary figures and images for: Pomacea canaliculata hemocyanin as a novel natural immunostimulant in mammals
Source: Front Immunol. 2025 Jan 8;15:1490260. doi: 10.3389/fimmu.2024.1490260 (PMC11750813; doi:10.3389/fimmu.2024.1490260)

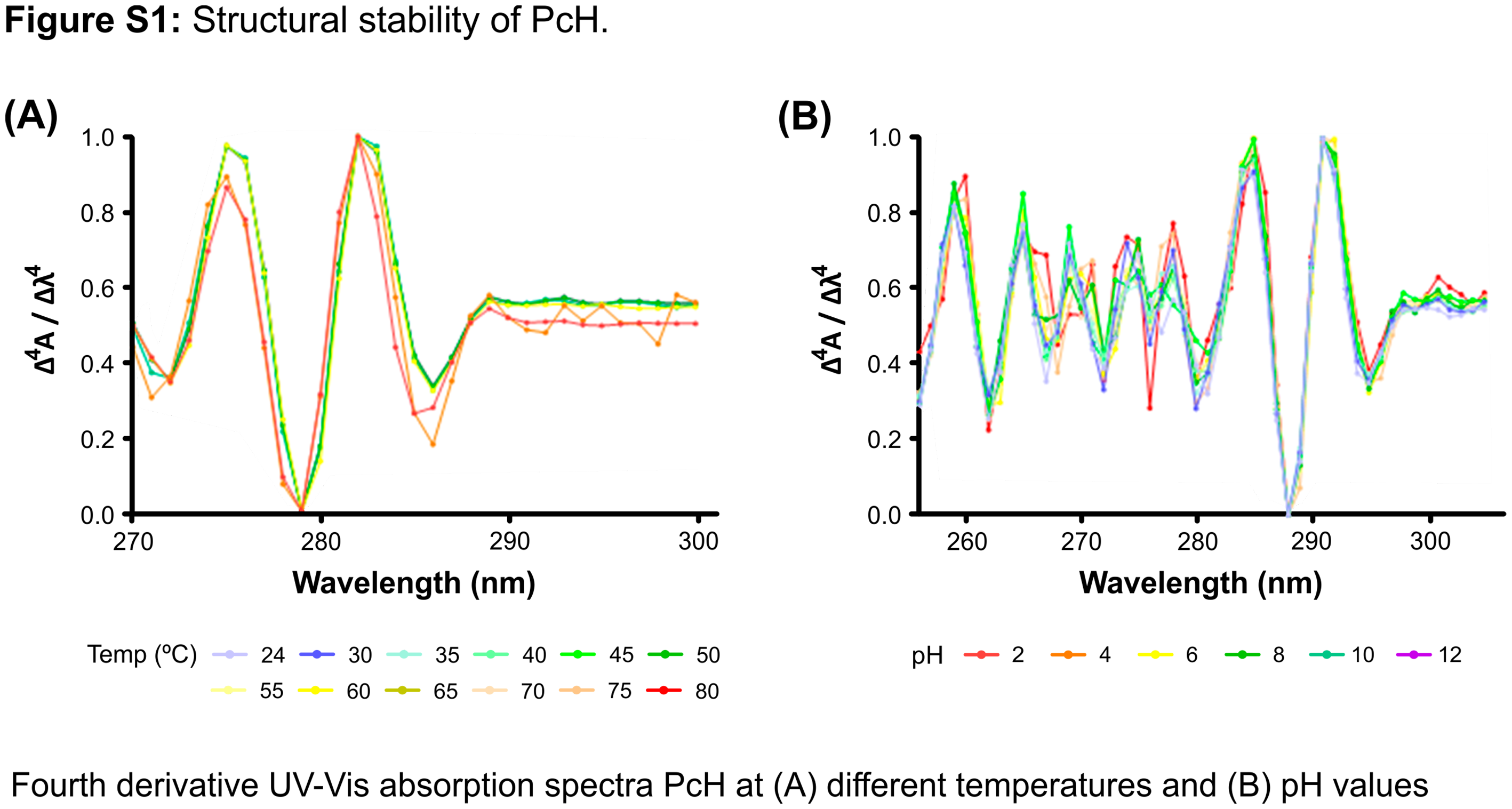

Supplement: Supplementary file 1 [file Image1.tif]

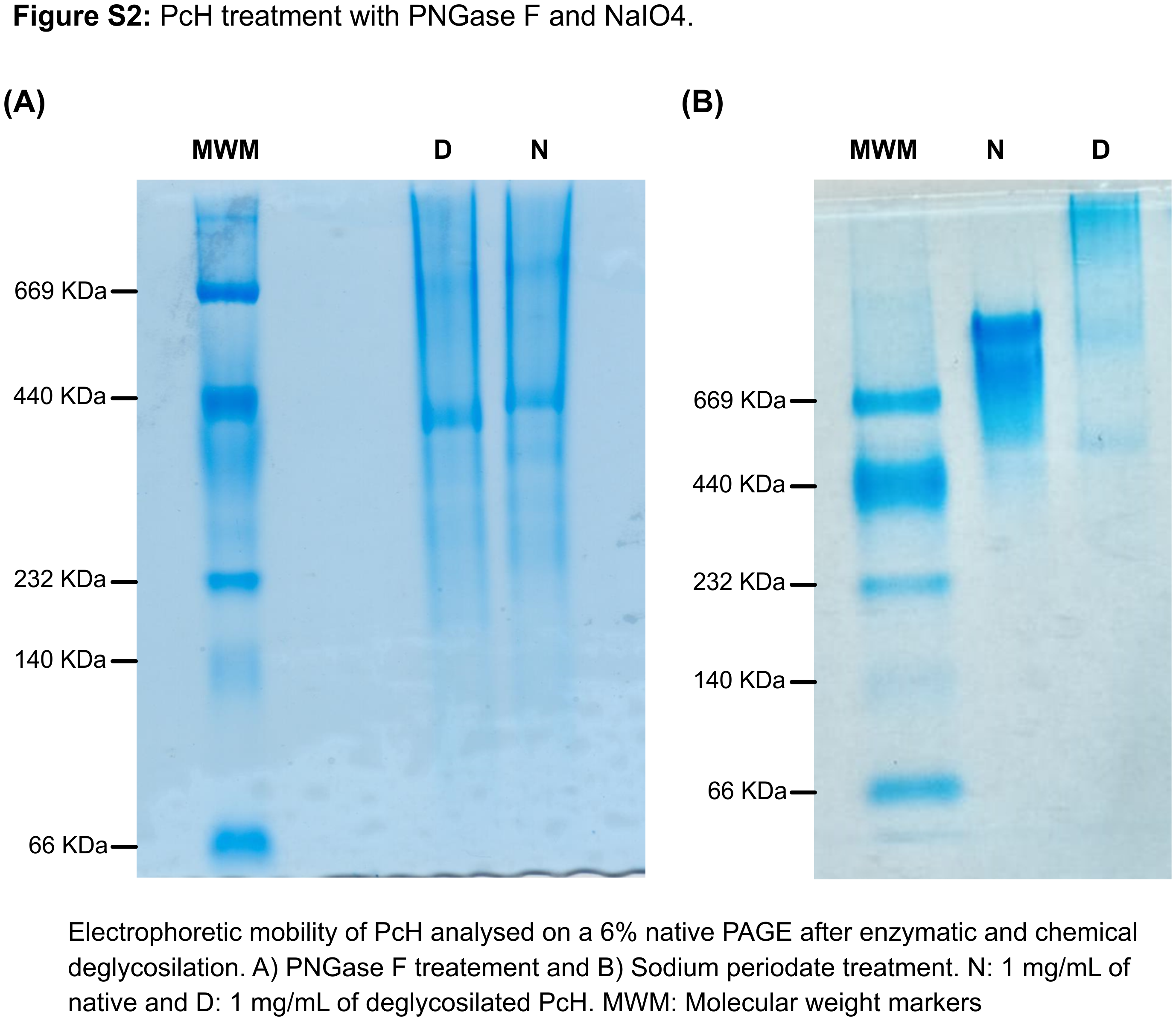

Supplement: Supplementary file 2 [file Image2.tif]
